# An AIEgen-based 3D covalent organic framework for white light-emitting diodes

**DOI:** 10.1038/s41467-018-07670-4

**Published:** 2018-12-07

**Authors:** Huimin Ding, Jian Li, Guohua Xie, Guiqing Lin, Rufan Chen, Zhengkang Peng, Chuluo Yang, Baoshan Wang, Junliang Sun, Cheng Wang

**Affiliations:** 1 Key Laboratory of Biomedical Polymers (Ministry of Education), College of Chemistry and Molecular Sciences, Wuhan University, Wuhan, 430072 China; 2 College of Chemistry and Molecular Engineering, Beijing National Laboratory for Molecular Sciences, Peking University, Beijing, 100871 China; 3 Department of Materials and Environmental Chemistry, Stockholm University, Stockholm, 10691 Sweden

## Abstract

The design and synthesis of three-dimensional covalent organic frameworks (3D COFs) have still been considered as a big challenge. Here we report the design and synthesis of an AIEgen-based 3D COF (3D-TPE-COF), with a high surface area (1084 m^2^ g^−1^). According to powder X-ray diffraction and continuous rotation electron diffraction analyses, 3D-TPE-COF is identified to adopt a seven-fold interpenetrated **pts** topology. Interestingly, 3D-TPE-COF emits yellow fluorescence upon excitation, with a photoluminescence quantum yield of 20%. Moreover, by simply coating 3D-TPE-COF onto a commercial blue light-emitting diode (LED), a prototype white LED (WLED) under continuously driving without degradation for 1200 h was demonstrated. The present work suggests the possibility of using COF materials for stable WLEDs, which will greatly inspire us to design and synthesize fluorescent 3D COFs and facilitate the development of COF-based WLEDs in future.

## Abstract (web summary)

3D covalent organic frameworks (COF) show interesting hierarchical arrangements of nanopores and open sites but their synthesis remains challenging. Here the authors report a fluorescent AIEgen-based 3D COF and demonstrate application as a coating material for white LEDs and for sensing of picric acid

## Introduction

Covalent organic frameworks (COFs) represent an emerging class of crystalline porous polymers with periodic two- or three-dimensional (2D or 3D) structure^1–4^. Due to their permanent porosity, high stability, and designable functionality, COFs have gained considerable attention as promising applications in gas storage and separation^5,6^, catalysis^7–10^, sensing^11–13^, optoelectronics^14–17^, energy storage^18–21^, etc. For all these reported systems, most of the examples are focusing on 2D COFs, and their synthesis and characterization have been relatively well established^22–27^. By contrast, since Yaghi and co-workers reported the first example in 2007^28^, only a few 3D COFs have been announced over the past decade^29–39^, mainly due to their limited availability of molecular building blocks and synthetic challenge. However, it should be emphasized here, as these 3D architectures can allow hierarchical arrangement of nanopores and possess numerous open sites, some reported 3D COFs have shown interesting applications, for example, size-selective catalysis^36^. Considering their unique structures and properties, the rational design and synthesis of 3D COFs with specific functions are highly demanded.

Aggregation-induced emission (AIE) is a novel photophysical phenomenon in which the luminogens are non-emissive in dilute solution but become highly emissive in aggregate state^40,41^. Since firstly coined by Tang and co-workers in 2001^42^, AIE luminogens (AIEgens) based materials have gained intensive attentions and shown broad applications in light-harvest^43^, light-emission^44^, chemo- and biosensors^45,46^, bioimaging^47^, and so on. The construction of AIEgen-based 3D COFs, which will precisely integrate AIEgens into 3D porous crystalline organic framework, should be very interesting^48,49^. First, the rigid skeleton can restrict the intramolecular rotation, vibration, and motions of AIEgens. Consequently, the obtained 3D COFs may be fluorescent, which can provide a unique platform for us to understand the underlying mechanism of AIE. Second, as solid-state fluorescent porous materials, these 3D COFs may find interesting applications in different areas, for example, sensing and optoelectronic devices. However, due to the synthetic challenge, AIEgen-based 3D COFs have not been reported yet.

Recently, we reported the design and synthesis of 3D COFs bearing pyrene^29^ or porphyrin^30^ units, starting from tetrahedral and quadrilateral building blocks connected through [4 + 4] imine condensation reactions. According to these results, we believe it is reasonable to construct other 3D COFs via this topology design. Therefore, we decided to choose the representative AIEgens, tetraphenylethylene (TPE)^41^, as the quadrilateral core to synthesize AIEgen-based 3D COFs.

**Fig. 1:**
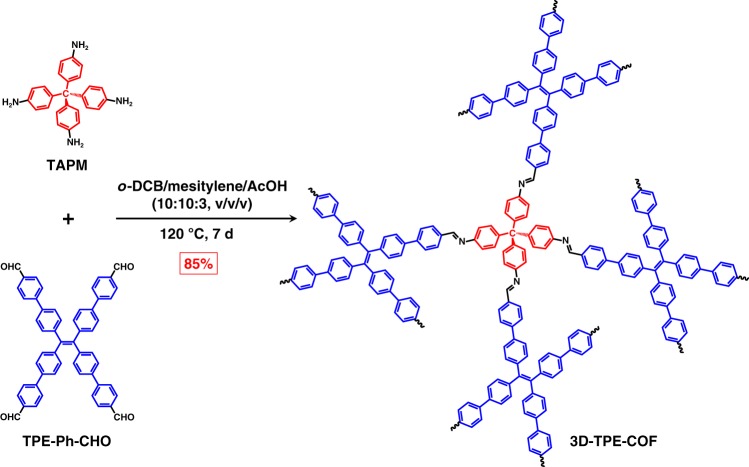
Chemical Structure. Schematic representation of the synthesis of 3D-TPE-COF

In this work, we reported the design and synthesis of a TPE-based 3D COF, named 3D-TPE-COF (Fig. 1). Our result clearly shows that the 3D-TPE-COF is a microporous material with high surface area, and by using the newly developed continuous rotation electron diffraction (cRED) method, 3D-TPE-COF is identified to adopt a seven-fold interpenetrated **pts** (the net of the platinum and sulfur atoms in PtS) topology with *P*2/*c* space group. Interestingly, 3D-TPE-COF emits yellow fluorescence upon excitation, with a photoluminescence quantum yield (PLQY) of 20%. Moreover, by simply coating 3D-TPE-COF onto a commercial blue light-emitting diode (LED), a prototype white LED (WLED) was fabricated. The Commission International de I’Eclairage (CIE) coordinates of COF-based WLED were determined to be (0.30, 0.35), which are close to that of the pure white light. In addition, this WLED exhibited no degradation after aging for 1200 h at ambient condition.

## Results

### Synthesis and characterization of 3D-TPE-COF

3D-TPE-COF was synthesized by condensation of the reported tetra(*p*-aminophenyl)methane (TAPM)^50^ and 1,1,2,2-tetrakis(4-formyl-(1,1′-biphenyl))ethene (TPE-Ph-CHO)^51^ in a mixture of *o*-dichlorobenzene, mesitylene and 6 M aqueous acetic acid (10: 10: 3, v/v/v) at 120 °C for 7 days (Fig. 1). After exhaustively washed by Soxhlet extraction with tetrahydrofuran, acetone, and dichloromethane, 3D-TPE-COF was isolated in 85% yield as a yellow powder insoluble in common organic solvents and water. From the Fourier transform infrared (FT-IR) spectrum, 3D-TPE-COF exhibited the diagnostic vibrational peak of the imine bond at 1625 cm^−1^ (Supplementary Figure 2), which is the same as that of the model compound (Supplementary Figure 1). In addition, the ^13^C cross-polarization with total suppression of sidebands (CP-TOSS) NMR spectrum of 3D-TPE-COF also confirmed the formation of the expected C=N bond at 158.3 ppm (Supplementary Figure 3). Furthermore, field-emission scanning electron microscopy images showed that 3D-TPE-COF has a rod-like morphology (Supplementary Figure 4), consisting of microcrystals.

**Fig. 2:**
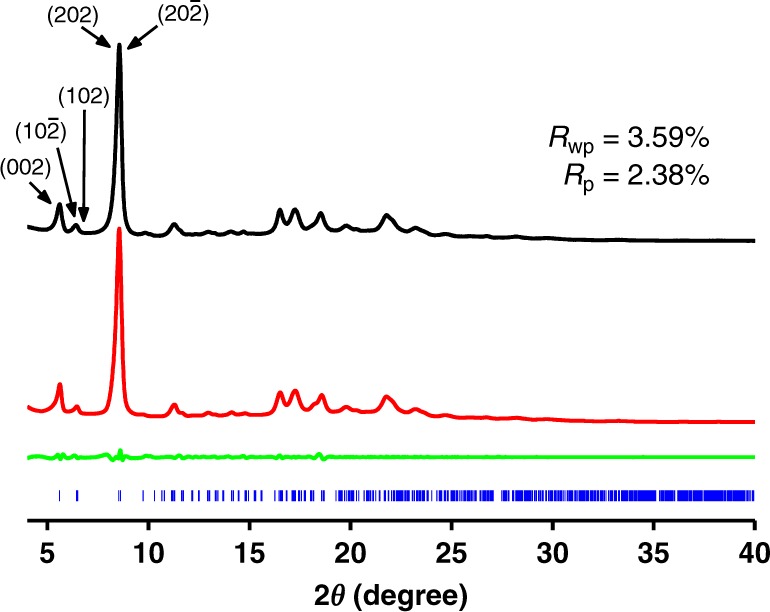
Powder X-ray diffraction patterns of 3D-TPE-COF. PXRD profiles of experimental pattern (black curve), Le Bail fitting pattern (red curve), their difference (green curve) and Bragg position from the seven-fold interpenetrated **pts** structure (blue curve)

**Fig. 3:**
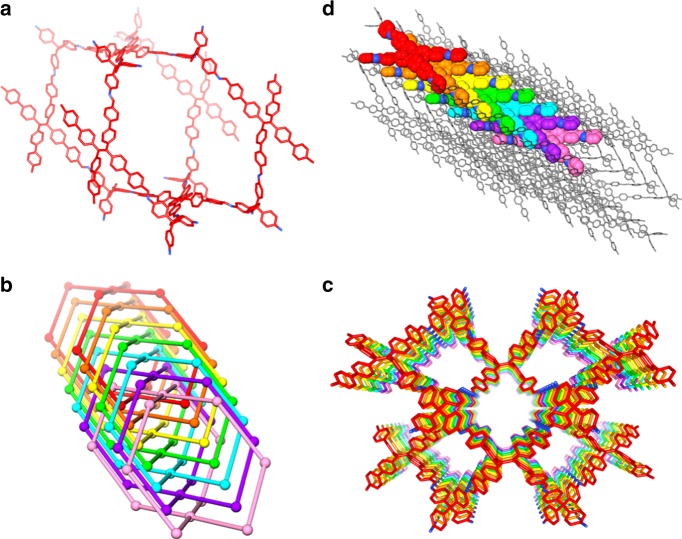
Structural representations of 3D-TPE-COFs. **a** Single **pts** network; **b** seven-fold interpenetrated **pts** topology; **c** the porous structure of 3D-TPE-COF; **d** the stacking behavior of TPE molecules in framework. Hydrogen atoms are omitted

The powder X-ray diffraction (PXRD) experiment was performed to demonstrate the crystalline nature of 3D-TPE-COF. As shown in Fig. 2, the experimental PXRD pattern displayed amounts of intense diffraction peaks, indicating the presence of a long-range ordered structure. However, it was very difficult to determinate the structure from PXRD data, due to the serious overlap of diffraction peaks. Since the nano- and micrometer-sized crystals can be treated as single crystal in electron crystallography, RED technique has recently been applied as a powerful tool for the structure determination of 3D COFs with **dia** topology^31–33^. The newly developed technique, cRED^52^, was then used to collect 3D electron diffraction, in which the time for data collection can be reduced to less than 5 min (see Supplementary method 3 for details). After carefully analysing the cRED data, we successfully located the central carbon atoms of tetrahedral and quadrilateral building blocks (Supplementary Figure 8) by *SHELXT*^53^. Based on the atom coordinates determined from cRED data, we were able to build a structure model with seven-fold interpenetrated **pts** net (Fig. 3) using the Materials studio software package, in which the calculated PXRD patterns of optimized model matched well with the experimental data (Fig. 2 and Supplementary Figure 9). The Le Bail fitting was performed on the experimental PXRD of 3D-TPE-COF, which yields a unit cell with parameters (*a* = 27.331(1) Å, *b* = 8.543(5) Å, *c* = 31.508(0) Å, *α* = *γ*  = 90°, and *β* = 90.476(0)°, *P*2*/c*). The final *R*_wp_ and *R*_p_ values converged to 3.59 and 2.38%, respectively. Therefore, 3D-TPE-COF is identified to adopt a seven-fold interpenetrated **pts** topology with *P*2/*c* space group.

**Fig. 4:**
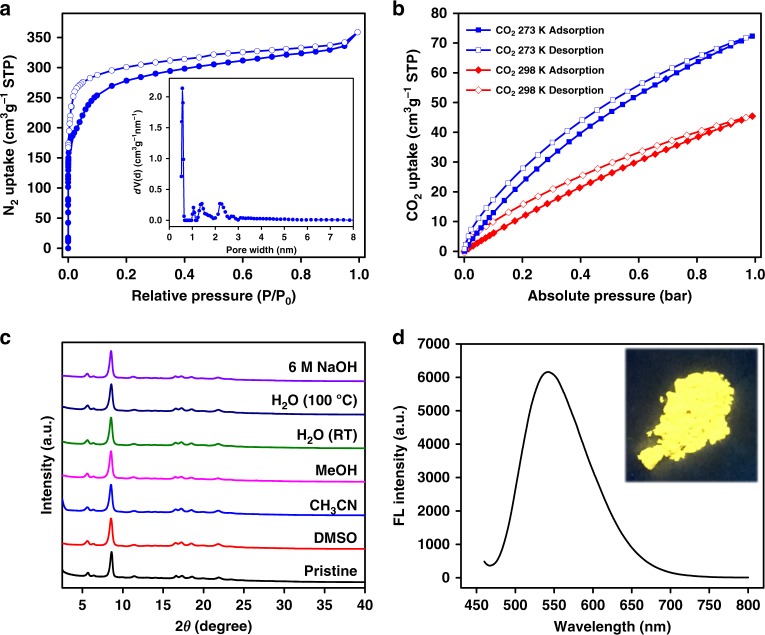
Characterization of 3D-TPE-COF. **a** N_2_ adsorption and desorption isotherms of 3D-TPE-COF at 77 K. The inset is the pore size distribution of 3D-TPE-COF calculated from the quenched solid density functional theory; **b** CO_2_ adsorption and desorption isotherms of 3D-TPE-COF at 273 K and 298 K; **c** PXRD patterns of 3D-TPE-COF after treatment in different solvents for 24 h; **d** solid-state fluorescence spectrum of 3D-TPE-COF (λ_ex_ = 450 nm). The inset is the photograph of 3D-TPE-COF powders under UV light irradiation

The permanent porosity of 3D-TPE-COF was determined by N_2_ adsorption measurement at 77 K. As shown in Fig. 4a, 3D-TPE-COF showed a type I isotherm, which is characteristic of microporous materials. The Brunauer–Emmett–Teller (BET) surface area and total pore volume were calculated to be 1084 m^2^ g^−1^ and 0.55 cm^3^ g^−1^, respectively. It is worth noting that this BET surface area is much higher than that of its amorphous analog (512 m^2^ g^−1^)^51^, which is also synthesized from TAPM and TPE-Ph-CHO. The pore width distribution calculated by the quenched solid density functional theory (QSDFT) model revealed one major peak centered at 0.57 nm (Fig. 4a). We also studied the CO_2_ adsorption behavior of 3D-TPE-COF (Fig. 4b), and the uptake value was 72 cm^3^ g^−1^ at 273 K and 45 cm^3^ g^−1^ at 298 K. In addition, the thermogravimetric analysis revealed that 3D-TPE-COF starts to decompose at 400 °C (see Supplementary Figure 6), indicating the high thermal stability. More importantly, this COF showed good chemical stability (Fig. 4c). After dispersing 3D-TPE-COF in common organic solvents, water and alkaline solution for 24 h, the COF samples still exhibited strong diffraction peaks in PXRD patterns. This is different from the reported boronate-linked 2D TPE-Ph COF^12^, which will lose crystalline structure upon exposure to water.

Considering the unique photophysical phenomenon of TPE chromophore, we then investigated the fluorescent properties of 3D-TPE-COF. Before that, we studied the fluorescence spectrum of the model compound in solid state. As shown in Supplementary Figure 12, the powder of the model compound exhibited yellow-green emission peaking at 534 nm upon excitation. However, the PLQY of the model compound is considerably low (6.6%), as the photo-induced electron transfer from TPE units to imine groups will quench the fluorescence^54^. Interestingly, 3D-TPE-COF can emit yellow light with an emission maximum at 543 nm (Fig. 4d), but with a much higher PLQY (20%). According to the crystal structure (Fig. 3d), we believe this enhancement can be ascribed to the aggregation of TPE units in the 3D framework. In addition, the rigid skeleton can restrict the relaxation of TPE units, which may also contribute this enhancement. Due to the fluorescent and porous nature of 3D-TPE-COF, we tested its chemosensing behavior by choosing the commercially available picric acid (PA) as explosive model (Supplementary Figure 14) in water. Obviously, the fluorescence of 3D-TPE-COF was quenched when PA was gradually added into the suspension, and the Stern–Volmer curve quenching constant (*K*_sv_) was estimated to be 3.3 × 10^4^ M^‒1^.

### Utilizing 3D-TPE-COF as coating material for WLED

**Fig. 5:**
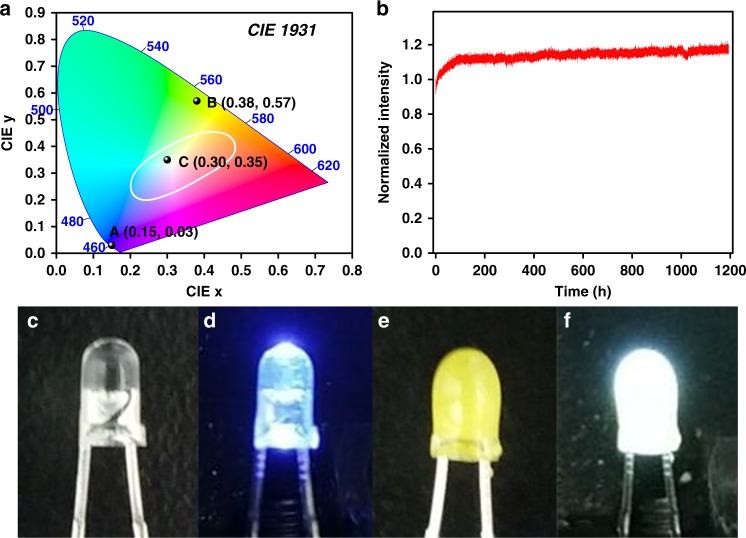
Characterization and photographs of the LEDs. **a** CIE-1931 chromaticity diagram and the positions (marked by the dots) for blue LED (0.15, 0.03; **a** 3D-TPE-COF (0.38, 0.57; **b** (λ_ex_ = 450 nm), and COF-coated LED (0.30, 0.35; (**c**). **b** Normalized luminescence intensity vs. time curve of the 3D-TPE-COF-coated WLED driven at 2 mA. Photographs of the LEDs: **c** a reference blue LED (not turned on), **d** the reference blue LED turned on (emission with blue light), **e** the same LED coated with a thin layer of 3D-TPE-COF (not turned on), and **f** the coated LED turned on (emission with white light)

WLEDs have attracted extensive attention due to their wide applications in display and lighting systems^55–58^. A common approach to fabricate WLEDs is coating a yellow-emitting phosphor on a blue LED chip, and the white light can be produced from the combined down-converted emissions of the phosphor and the blue component of the LED chip. As 3D-TPE-COF emits yellow fluorescence upon excitation with blue light, we then explore the possibility of using this 3D COF for WLEDs. By using a simple dip-coating procedure (see Supplementary Method 6 for details), a thin film of 3D-TPE-COF can be homogeneously coated onto the surface of a commercially available blue LED lamp (peaking around 450 nm). As shown in Fig. 5, when the LED was turned on, bright white light was generated. The CIE coordinates were determined to be (0.30, 0.35), which are close to the standard coordinates for pure white light (0.33, 0.33). To explore the potential of practical application, we further tested the stability of this prototype WLED (Fig. 5b). Obviously, the luminescence of the COF-coated WLED is considerably stable under continuously driving at ambient condition for 1200 h. Such highly stable WLED has seldom been reported for the one with a down-conversion layer of organic compounds. Therefore, by simply coating onto the commercial blue chips, 3D-TPE-COF has shown interesting application in robust, low-cost, and rare-earth metal-free WLEDs.

## Discussion

We have reported the design and synthesis of an AIEgen-based 3D COF, 3D-TPE-COF, starting from tetrahedral and quadrilateral building blocks connected through [4 + 4] imine condensation reactions. The obtained 3D-TPE-COF was characterized by several techniques, and according to cRED analyses, it is identified to adopt a seven-fold interpenetrated **pts** topology with *P*2/*c* space group. Interestingly, the immobilization of TPE into 3D COF can allow the resulting 3D-TPE-COF emit yellow fluorescence upon excitation. Moreover, by simply coating 3D-TPE-COF onto a commercial blue LED, the COF-based WLED was constructed with the CIE coordinates of (0.30, 0.35), which showed no degradation under continuously driving at ambient condition for 1200 h. The present work demonstrates that fluorescent 3D COFs is feasible to fabricate rare-earth metal-free WLEDs, which will inspire us to facilitate the development of other fluorescent 3D COFs with higher quantum yields. In addition, considering the intrinsic porosity, the accommodation of dye molecules within the pores of fluorescent 3D COFs may also offer another strategy to construct WLEDs. Currently, these studies are undergoing in our lab.

## Methods

### Materials

All the reagents and starting materials were purchased from Acros, Adamas or TCI, and used without further purification unless otherwise noted. Dehydrated solvents were obtained after treating solvents with standard procedures. Tetrakis-(4-aminophenyl)methane (TAPM)^50^ and 1,1,2,2-tetrakis(4-formyl-(1, 1′-biphenyl))ethene (TPE-Ph-CHO)^51^ were synthesized according to the literature. The blue LED [https://item.jd.com/12630843985.html] was purchased online.

### Characterizations

High-resolution mass spectra were collected on DIONEX UltiMate 3000 & Bruker Compact TOF and Bruker Solarix mass spectrometers. Solid-state NMR spectra were recorded at ambient temperature on a Bruker AVANCE III 400M spectrometer. Fourier transform infrared (FT-IR) spectra were recorded on a Nicolet iN10 micro FTIR Spectrometer. Elemental analysis was conducted on a Flash EA 1112. The PXRD patterns were obtained on a Rigaku Smartlab X-ray diffractometer with Cu K*α* line (λ = 1.54056 Å). Thermogravimetric analysis was performed on a TGA-Q500 from 30 to 800 °C at the rate of 10 °C min^−1^ without equilibration delay. Field-emission scanning electron microscopy (FE-SEM) images were performed on a Zeiss ∑IGMA operating at an accelerating voltage ranging from 0.1 to 20 kV. The samples were prepared by dispersing the material onto conductive adhesive tapes attached to a flat aluminum sample holder and then coated with gold. Nitrogen isotherms were measured at 77 K using an Autosorb-iQ (Quantachrome) surface area size analyzer.

The 3D electron diffraction data were collected on a 200 kV JEOL JEM-2100 transmission electron microscope, which was equipped with a quad hybrid pixel detector (Timepix). For data collection, a micro single crystal was selected and placed in the electron beam, and then the Z height was adjusted to the mechanical eucentric height. The selected-area ED patterns were captured from the crystal continuously when the goniometer was rotated. Finally, 374 ED patterns were recorded and the tilt rang was from −52.29° to 34.3° with the tilt step of 0.23°. The total time for data collection was 191 s. UV-Vis spectra were recorded on a SHIMADZU UV-3600 UV-vis-NIR spectrophotometer. Fluorescence spectra were recorded on a HITACHI F-4600 spectrofluorometer. Absolute quantum yields were obtained using a Quantaurus-QY measurement system (C9920–02, Hamamatsu Photonics) and all the samples were excited at 450 nm. A PR735 SpectraScan Spectroradiometer (Photo Research) was used to simultaneously record the electroluminescent spectra and CIE coordinates. The intensity *vs*. time curve of the COF-coated LED driven at 4 mA was recorded by a lifetime measurement system (Crysco) at room temperature.

### Synthesis of 3D-TPE-COF

A Pyrex tube was charged with TAPM (25.5 mg, 0.067 mmol), TPE-Ph-CHO (50 mg, 0.067 mmol), 2.0 mL *o*-dichlorobenzene and 2.0 mL mesitylene. The mixture was sonicated for 10 min, followed by slow addition of 0.6 mL of 6 M aqueous acetic acid. After that, the tube was degassed by three freeze−pump−thaw cycles, sealed under vacuum and then heated at 120 °C for 7 d. The reaction mixture was cooled to room temperature and the resulting precipitate was filtered off, exhaustively washed by Soxhlet extractions with tetrahydrofuran, acetone, and dichloromethane for 24 h. The resulting yellow powder was dried at 80 °C under vacuum for overnight to give 3D-TPE-COF in 85% yield. Elemental analysis for the calculated: C, 89.74%; H, 4.96%; N, 5.30%. Found: C, 84.58%; H, 5.28%; N, 4.85 %.

### Fabrication of COF-coated WLED

The activated 3D-TPE-COF (~10 mg) was loaded into an agate mortar and manually ground with the pestle for 10 min to afford a fine powder. After that, 0.5 mL UV curable epoxy (Norland NOA 63) was then added to the mortar, and the mixture was stirred until a homogeneous slurry was achieved. By using a simple dip-coating procedure, the mixture was uniformly dispersed on the surface of a commercially available blue LED (peaking at ~450 nm), which matched well with the absorption of 3D-TPE-COF. This coating process can be easily repeated to ensure an even and continuous coating of 3D-TPE-COF on the dome of the blue LED. After irradiation under UV lamp (SMC-400H, Sunwheel Materials Co., Ltd) for 2 min, the outer layer was then covered with a high-quality UV curable epoxy (ACW A90535-AN) which possessed a low water permeability down to 2.7 × 10^−4^ g m^−2^ per day. Finally, the COF-coated WLED was obtained and tested at ambient conditions after curing the epoxy again for 2 min.

## Electronic supplementary material

Supplementary Information

## Data Availability

All the data supporting the findings of this study are available within the Article and its Supplementary Information, or from the corresponding author (C.W. or J.S.) upon reasonable request.
