# Supplementary material for: An AIEgen-based 3D covalent organic framework for white light-emitting diodes
Source: Nat Commun. 2018 Dec 7;9:5234. doi: 10.1038/s41467-018-07670-4 (PMC6286360; doi:10.1038/s41467-018-07670-4)
Supplement: Supplementary file 1 — Supplementary Information [file 41467_2018_7670_MOESM1_ESM.pdf]

Supplementary Information

An AIEgen-based 3D Covalent Organic Framework for White Light-emitting Diodes – Ding *et al*.

Supplementary Method 1

Synthetic Procedures

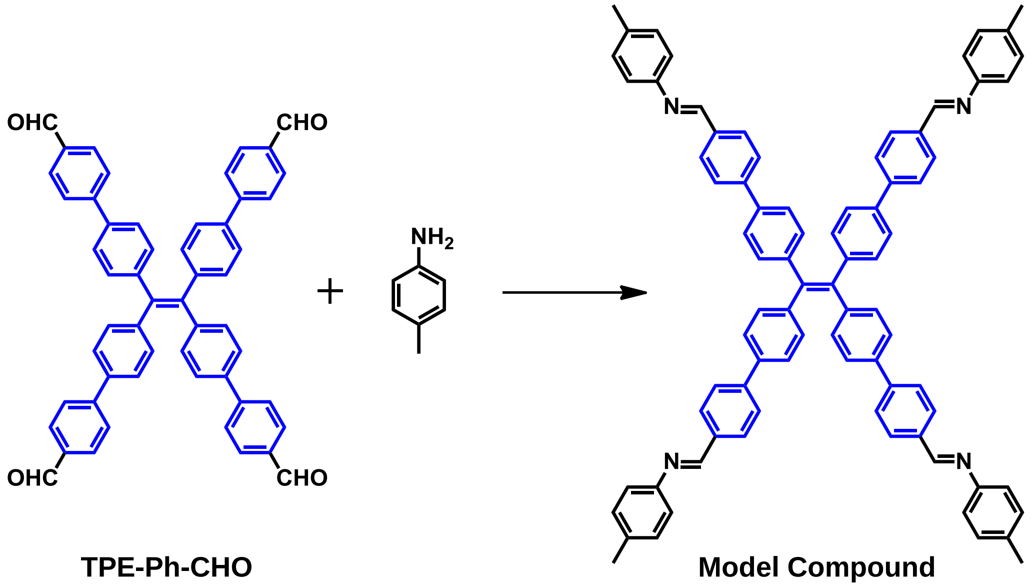

Supplementary Figure 1 | Synthesis of model compound.

Synthesis of model compound: TPE-Ph-CHO (100 mg, 0.134 mmol), *p*-toluidine (80 mg, 0.747 mmol), acetic acid (50 μL) was added to a 50 mL flask containing dichloromethane (10 mL) and ethanol (10 mL). The mixture was degassed and then allowed to reflux for 4 hours under N_2. After cooling down to room temperature, a large amount of yellow solid precipitated out from the solution. The precipitate was filtered and washed with ethanol and finally dried in vacuum. The product was isolated as yellow solid (138 mg, yield: 94 %). ^{1}H NMR (400 MHz, $CDCl_3$, 298 K, ppm): δ 8.49 (s, 4H), 7.94 (d, *J* = 8.4 Hz, 8H), 7.71 (d, *J* = 8.4 Hz, 8H), 7.50 (d, *J* = 8.3 Hz, 8H), 7.24 – 7.20 (m, 16H), 7.17 (d, *J* = 8.4 Hz, 8H), 2.38 (s, 12H). ^{13}C NMR (100 MHz, $CDCl_3$, 298K, ppm): δ 159.1, 149.4, 143.3, 143.1, 140.5, 138.3, 135.8, 135.2, 132.1, 129.8, 129.2, 127.1, 126.5, 120.8, 21.0. HR-MS: calcd for $C_{82}H_{65}N_4$ m/z = 1105.5209 $[M+H]^+$, found: m/z = 1105.5189 $[M+H]^+$.

Synthesis of TPE-Ph-CHO: It was synthesized according to the literature [1] with some modification. Tetrakis(4-bromophenyl)ethylene (644 mg, 1 mmol),

4-formylphenylboronic acid (1.35 g, 9 mmol), K_2CO_3 (1.4 g, 10 mmol) and $Pd(PPh_3)_4$ catalyst (58 mg, 0.05 mmol) was added to a flask containing 1, 4-dioxane (30 mL). The mixture was refluxed under nitrogen for 24 h. After cooling to room temperature, the solvents were evaporated under reduced pressure and the resulting residue was subjected to column chromatography. The mixture was purified by column chromatography [SiO_2 (200-300): PE / CH_2Cl_2 (2: 3, by vol.) →PE / CH_2Cl_2 /EtOAc (20: 30: 1, by vol.)] to give a yellow solid (516 mg, yield, 69%). ^{1}H NMR ($CDCl_3$, 400 MHz, 298 K, ppm): δ = 10.03 (s, 1H), 7.92 (d, J = 8.3 Hz, 2H), 7.74 (d, J = 8.3 Hz, 2H), 7.48 (d, J = 8.4 Hz, 2H), 7.23 (d, J = 8.4 Hz, 2H). ^{13}C NMR ($CDCl_3$, 100 MHz, 298 K, ppm): δ = 192.0, 146.5, 143.7, 140.8, 135.3, 132.2, 127.5, 127.0, 77.4. HR-MS: calcd for $C_{54}H_{36}O_4$ m/z = 748.2614 $[M]^+$, found: m/z = 748.2604 $[M]^+$.

Synthesis of TAPM: It was synthesized according to the literature.[2] Tetrakis(4-nitrophenyl)methane (1.5 g, 2.99 mmol), hydrazine monohydrate (2.00 g, 40 mmol) and Raney-nickel (~10 g) were added into a flask containing THF (100 mL). After refluxing under nitrogen for 3 hours, the mixture was filtered and washed with THF. The resulting solution were then added with 2 mL of concentrated hydrochloric acid, which will lead to the formation of white precipitate. After that, the precipitate was collected and dissolved again in water. Finally, aqueous ammonia was added into the solution. The appeared white precipitates were filtered off and dried in vacuum, which gave the pure product (0.85 g, 2.23 mmol, 74.6 % yield). ^{1}H NMR (DMSO-d_6, 400 MHz, 298 K, ppm): δ = 6.67 (d, J = 7.6 Hz, 1H), 6.38 (d, J = 7.9 Hz, 1H), 4.85 (s, 1H). ^{13}C NMR (DMSO-d_6, 100 MHz, 298 K, ppm): δ = 145.7, 135.8, 131.1, 112.6, 61.1. HR-MS: calcd for $C_{25}H_{25}N_4$ m/z = 381.2079 $[M+H]^+$, found: m/z = 381.2063 $[M+H]^+$.

Supplementary Method 2

Characterization of 3D-TPE-COF

FT-IR Spectroscopy Analysis

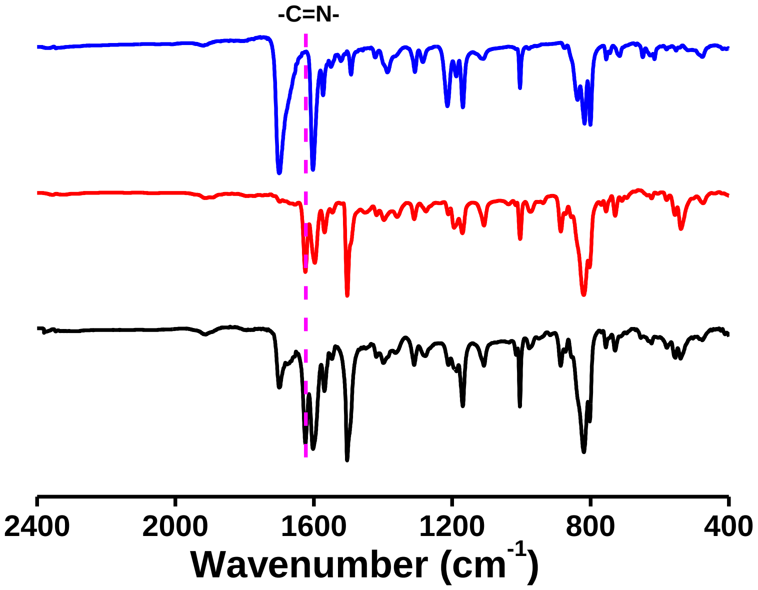

Supplementary Figure 2 | FT-IR spectra of TPE-Ph-CHO (blue curve), model compound (red curve) and 3D-TPE-COF (black curve). The appearance of a new band at 1625 cm^{-1} in 3D-TPE-COF confirmed the formation of imine linked C=N.

^{13}C Solid-State NMR Spectroscopy

High resolution solid-state NMR spectra were recorded at ambient pressure on a Bruker AVANCE III 400M spectrometer using a standard CP-TOSS pulse sequence (cross polarization with total suppression of sidebands) probe with 4 mm (outside diameter) zirconia rotors. Cross-polarization with TOSS was used to acquire ^{13}C data at 100.37 MHz. The ^{13}C ninety-degree pulse widths were 4 μs. The decoupling frequency corresponded to 72 kHz. The TOSS sample-spinning rate was 5 kHz. Recycle delays was 2s. The ^{13}C chemical shifts are given relative to glycine as 176.03 ppm. ^{13}C NMR of 3D-TPE-COF: δ = 158.3, 150.6, 143.3, 141.2, 135.2, 131.5, 128.1, 119.3, 64.5.

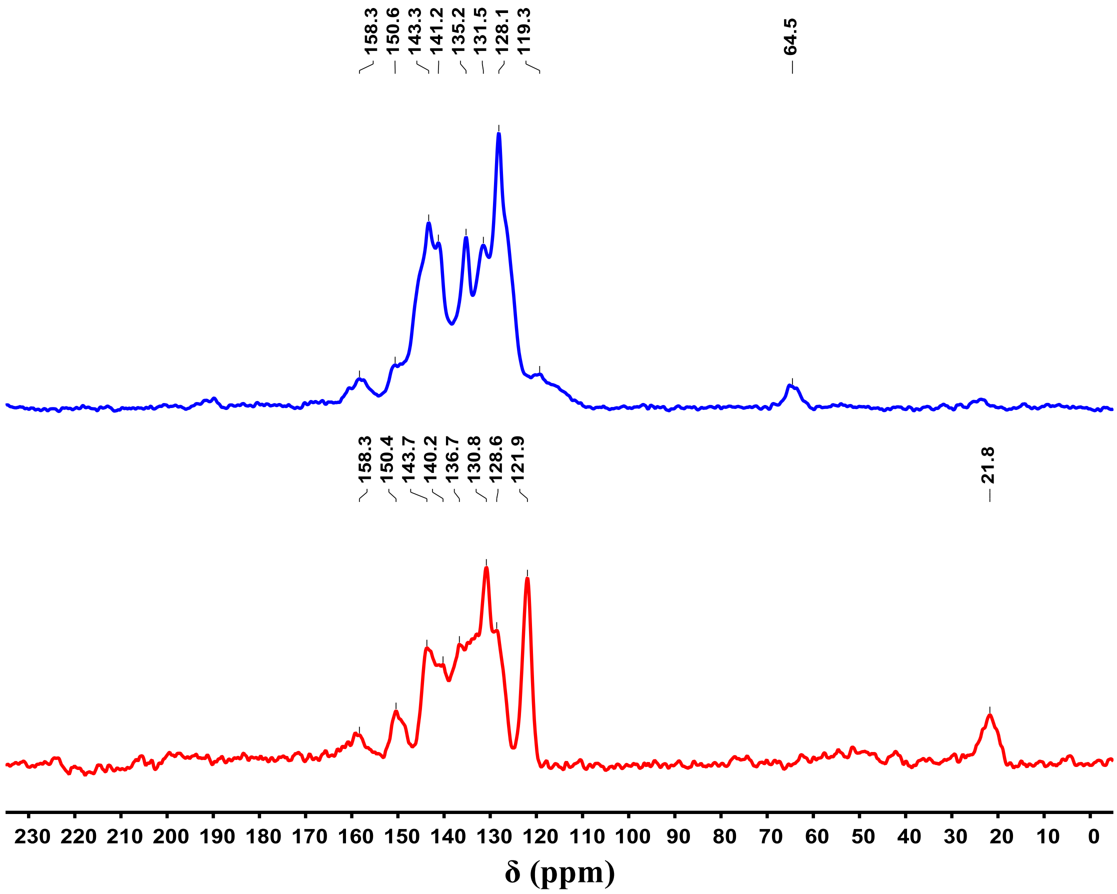

Supplementary Figure 3 | ^{13}C Solid-State NMR spectrum of model compound (red curve) and 3D-TPE-COF (blue curve).

Scanning Electron Microscopy Images

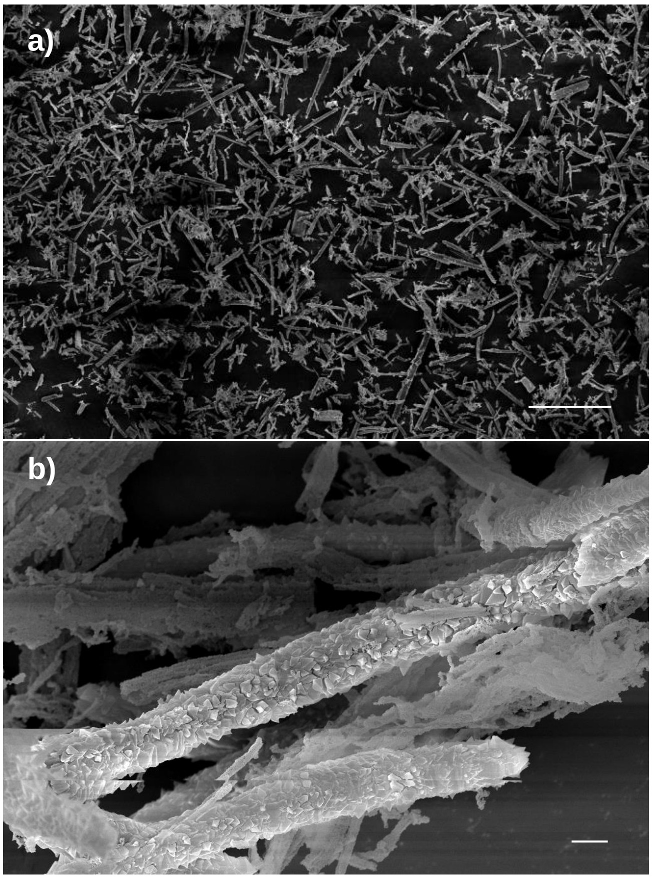

Supplementary Figure 4 | SEM images of 3D-TPE-COF. Scale bar: 100 μm (a), 2 μm (b).

Gas Sorption Isotherm measurements

Before measurement, the samples were degassed in vacuum at 150 °C for 12 h. For N_2 sorption isotherm measurements, a liquid N_2 bath was used for adsorption measurement at 77 K. To provide high accuracy and precision in determining P/P_0, the saturation pressure P_0 was measured throughout the N_2 analyses by means of a dedicated saturation pressure transducer, which allowed us to monitor the vapor pressure for each data point. The Brunauer-Emmett-Teller (BET) method was utilized to calculate the specific surface areas. To estimate pore size distributions for 3D-TPE-COF, the pore size distribution curve was analyzed using the quenched solid density functional theory (QSDFT) based on a carbon model containing slit/cylindrical. By using QSDFT adsorption branch model, the pore volume was

derived from the adsorption curve. The CO_2 sorption isotherms were measured up to 1 bar at 273 K and 298 K with precisely temperature control.

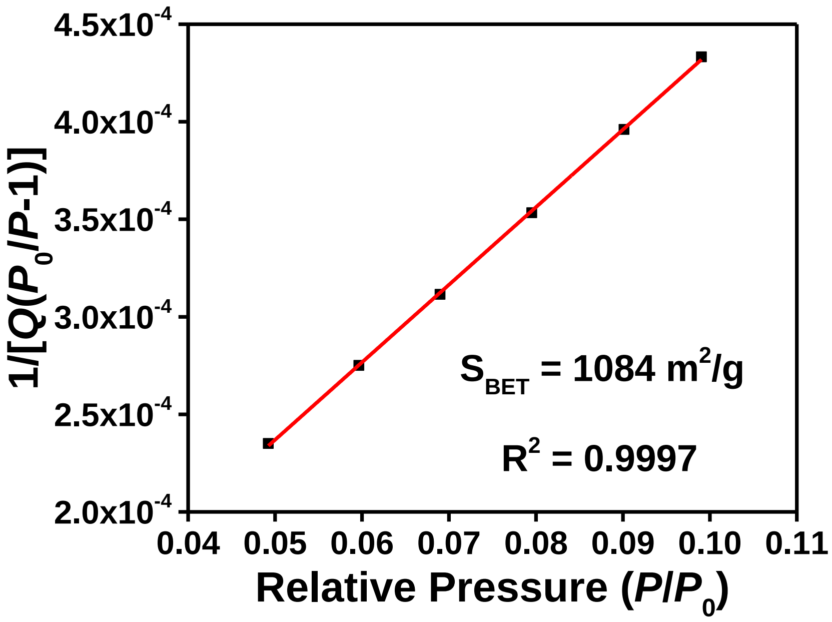

Supplementary Figure 5 | BET surface area plots for 3D-TPE-COF calculated from the N_2 adsorption isotherm at 77 K

Thermogravimetric Analysis

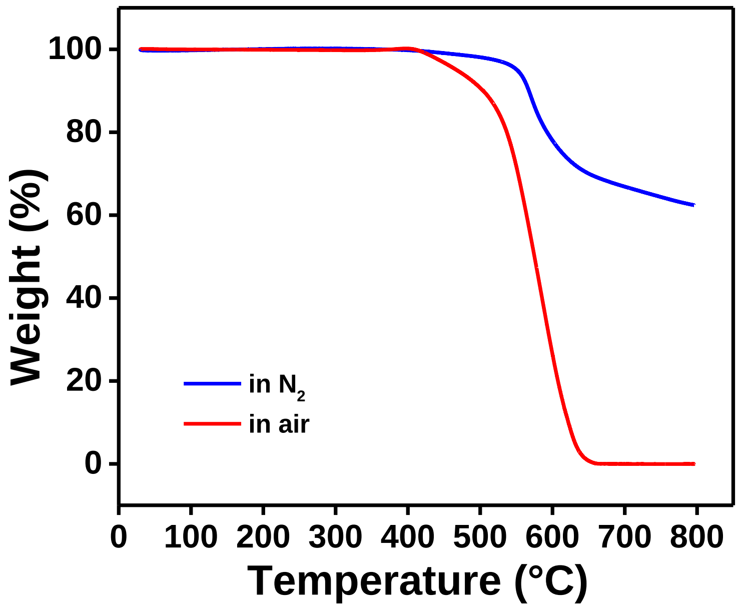

Supplementary Figure 6 | TGA profile of 3D-TPE-COF in N_2 (blue curve) and air (red curve)

Supplementary Method 3

Structure Analyses

The 3D electron diffraction data was collected on a 200kV JEOL JEM-2100 transmission electron microscope, which was equipped with a quad hybrid pixel detector (Timepix). For data collection, a micro single crystal was selected and placed in the electron beam, and then the Z height was adjusted to the mechanical eccentric height. The selected-area ED patterns were captured from the crystal continuously when the goniometer was rotated. In total, 374 ED patterns were recorded and the tilt rang was from -52.29° to 34.3° with the tilt step of 0.23°. The total time for data collection was 191 s (Supplementary Figure 7).

The cRED data set was processed by using the software REDp[3], including the origin shift, peak search, unit cell determination and indexing the reflection and intensity extraction. Finally, the hkl list with 264 unique reflections was obtained and ready for structure solution.

The structure of 3D-TPE-COF was then solved by using *SHELXT*[4]. Although the resolution of cRED data was only 2 Å, it was good enough to locate the central carbon atoms of tetrahedral and quadrilateral building blocks. With such rough model from cRED data, a model of seven-fold interpenetrated **pts** net was built under the symmetry of *P*2/*c* using Materials studio 7.0 software. The unit cell of the new model was optimized by performing Le Bail fitting on the experimental PXRD. The model of 3D-TPE-COF was further optimized using the universal force field (UFF) under the unit cell parameter that obtained from Le Bail fitting. The calculated PXRD patterns of optimized model matched well with the experimental data (Supplementary Figure 9), which confirmed the structure model of 3D-TPE-COF was reasonable.

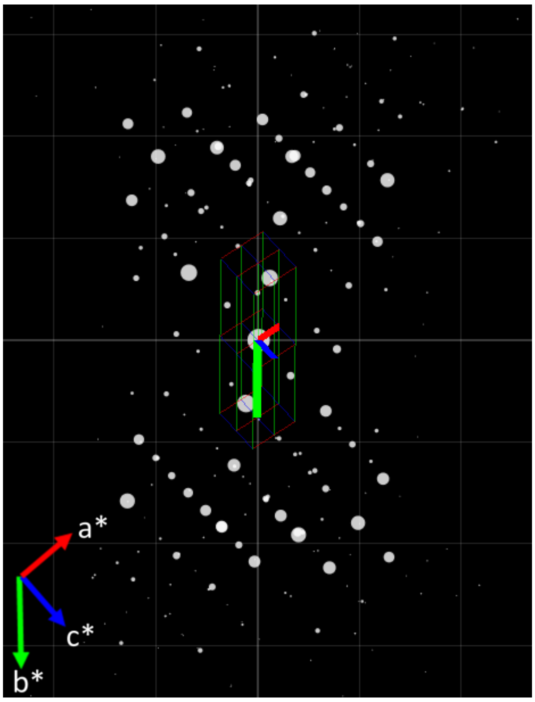

Supplementary Figure 7 | Overview of 3D reciprocal lattice of 3D-TPE-COF

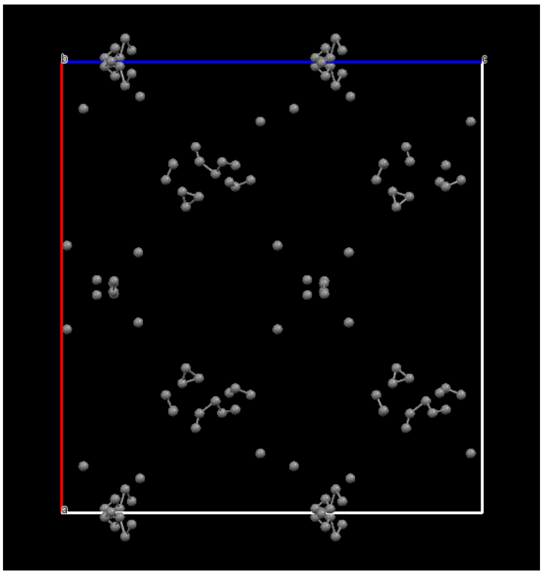

Supplementary Figure 8 | the original structure of 3D-TPE-COF obtained from cRED data

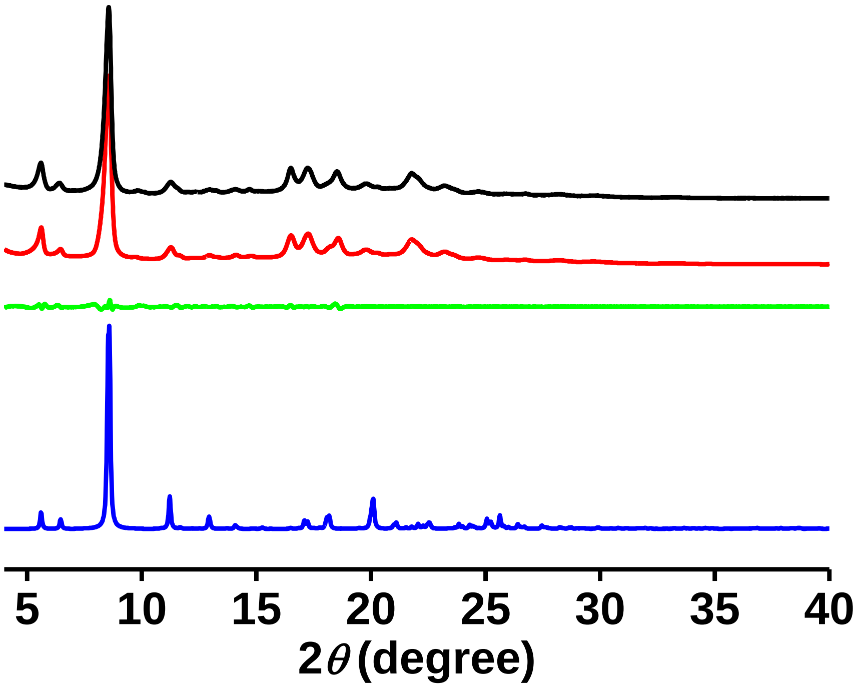

Supplementary Figure 9 | PXRD profiles of experimental pattern (black curve), Le Bail fitting pattern (red curve), their difference (green curve) and calculated patterns from seven-fold interpenetrated **pts** structure (blue curve).

Supplementary Table S1 | Fractional atomic coordinates for the unit cell of 3D-TPE-COF.

3D-TPE-COF		Space Group: *P*2/*c*<br>a = 27.331(1) Å, b = 8.543(5) Å, c = 31.508(0) Å,<br>$\alpha = \gamma = 90°$, and $\beta = 90.476(0)°$		
C1	C	0.53258	0.14133	0.77789
C2	C	0.57972	0.1952	0.7655
N3	N	0.60248	0.59105	0.82516
C4	C	0.51122	0.23714	0.81018
C5	C	0.57945	0.43572	0.81254
C6	C	0.53386	0.37736	0.82721
C7	C	0.60238	0.33473	0.78262
C8	C	0.4681	0.89359	0.7787
C9	C	0.43074	0.79567	0.76071
C10	C	0.42741	0.60584	0.82215
C11	C	0.48195	0.8458	0.8205

C12	C	0.4108	0.65853	0.78162
N13	N	0.41333	0.45263	0.84354
C14	C	0.4619	0.70963	0.84141
C15	C	0.96897	0.36818	0.77932
C16	C	0.92001	0.32099	0.7703
C17	C	0.92174	0.07972	0.81723
C18	C	0.99213	0.26949	0.8103
C19	C	0.89727	0.18413	0.78878
N20	N	0.89823	0.92632	0.83091
C21	C	0.9695	0.13196	0.8288
C22	C	0.03345	0.61626	0.77745
C23	C	0.06806	0.71872	0.75771
N24	N	0.09103	0.05794	0.8409
C25	C	0.02277	0.66178	0.81997
C26	C	0.07522	0.90642	0.8194
C27	C	0.04345	0.79896	0.84016
C28	C	0.08838	0.85735	0.77783
C29	C	0.2486	0.74922	0.51888
C30	C	0.41001	-0.30378	0.64506
C31	C	0.08711	1.81682	0.64102
C32	C	0.25774	0.7557	0.47357
C33	C	0.11953	1.82369	0.32753
C34	C	0.38071	-0.34118	0.33278
C35	C	0.15558	1.10469	0.54343
C36	C	0.22888	0.93803	0.58552
C37	C	0.17737	0.96626	0.52512
C38	C	0.21827	0.88477	0.54331
C39	C	0.20536	1.07331	0.60498
C40	C	0.16989	1.16939	0.58352
C41	C	0.08818	1.55607	0.59782
C42	C	0.17294	1.42948	0.63285
C43	C	0.10619	1.40559	0.58435
C44	C	0.1495	1.33437	0.6009
C45	C	0.15472	1.58226	0.6464
C46	C	0.11139	1.65216	0.62911
C47	C	0.15904	1.54491	0.32805
C48	C	0.14771	1.48488	0.41404
C49	C	0.17611	1.40052	0.3465
C50	C	0.17259	1.36573	0.39045
C51	C	0.12787	1.62681	0.39487
C52	C	0.13402	1.66445	0.35103
C53	C	0.3923	-0.11174	0.39208
C54	C	0.32011	0.07472	0.35422

C55	C	0.37917	0.04117	0.40933
C56	C	0.34246	0.14219	0.39102
C57	C	0.33301	-0.07649	0.33729
C58	C	0.3695	-0.17696	0.35514
C59	C	0.37461	0.0736	0.57783
C60	C	0.34763	-0.06046	0.65536
C61	C	0.39695	-0.06839	0.59332
C62	C	0.38385	-0.14385	0.63213
C63	C	0.32619	0.08552	0.64031
C64	C	0.33811	0.15627	0.60043
C65	C	0.19264	1.06066	0.39183
C66	C	0.25167	1.06417	0.46424
C67	C	0.21201	0.91822	0.41066
C68	C	0.24133	0.91186	0.44843
C69	C	0.22578	1.21114	0.44773
C70	C	0.19619	1.21149	0.41066
C71	C	0.27325	0.55564	0.40749
C72	C	0.34212	0.3722	0.44967
C73	C	0.29321	0.41465	0.38855
C74	C	0.32625	0.31019	0.40979
C75	C	0.32376	0.51735	0.46755
C76	C	0.28488	0.61004	0.4494
C77	C	0.24763	0.45093	0.53987
C78	C	0.33288	0.46378	0.58848
C79	C	0.27085	0.31149	0.55608
C80	C	0.3133	0.31158	0.58232
C81	C	0.31081	0.60419	0.57094
C82	C	0.26769	0.60258	0.54522
H83	H	0.59882	0.13265	0.74084
H84	H	0.47526	0.20613	0.82141
H85	H	0.51482	0.44084	0.85156
H86	H	0.63832	0.36611	0.77135
H87	H	0.41774	0.82318	0.72914
H88	H	0.51024	0.91065	0.83699
H89	H	0.38254	0.59349	0.76544
H90	H	0.47476	0.68247	0.8731
H91	H	0.8994	0.38733	0.74728
H92	H	1.02919	0.29721	0.81953
H93	H	0.86002	0.15693	0.77952
H94	H	0.99013	0.06586	0.85192
H95	H	0.07829	0.69415	0.72535
H96	H	-0.00367	0.59461	0.83764
H97	H	0.03302	0.82419	0.87248

H98	H	0.11428	0.92628	0.76029
H99	H	0.44036	-0.33451	0.62504
H100	H	0.05376	1.84142	0.62369
H101	H	0.13502	1.83574	0.29635
H102	H	0.35885	-0.3631	0.3047
H103	H	0.1278	1.16331	0.52475
H104	H	0.25698	0.87791	0.60349
H105	H	0.16271	0.92668	0.49501
H106	H	0.21685	1.10663	0.6366
H107	H	0.05475	1.59815	0.58308
H108	H	0.20645	1.38712	0.6473
H109	H	0.08494	1.34435	0.56054
H110	H	0.17499	1.64558	0.67053
H111	H	0.16665	1.56379	0.29484
H112	H	0.14333	1.46841	0.44785
H113	H	0.19457	1.31791	0.32609
H114	H	0.10976	1.71076	0.4151
H115	H	0.4206	-0.17879	0.40798
H116	H	0.29133	0.13779	0.33793
H117	H	0.39851	0.07988	0.43748
H118	H	0.31353	-0.11568	0.30918
H119	H	0.38625	0.11917	0.54748
H120	H	0.33587	-0.10697	0.68554
H121	H	0.42454	-0.12253	0.57371
H122	H	0.29952	0.14287	0.66015
H123	H	0.17355	1.05215	0.36195
H124	H	0.27769	1.07518	0.48997
H125	H	0.2028	0.81216	0.3949
H126	H	0.23343	1.32093	0.4628
H127	H	0.2474	0.61814	0.38833
H128	H	0.36927	0.30857	0.46787
H129	H	0.28093	0.38628	0.35683
H130	H	0.34146	0.55707	0.49614
H131	H	0.21415	0.43965	0.52166
H132	H	0.3671	0.47214	0.60522
H133	H	0.25578	0.20143	0.54685
H134	H	0.32936	0.71406	0.57492
C135	C	0.5	0.01687	0.75
C136	C	0	0.49202	0.75

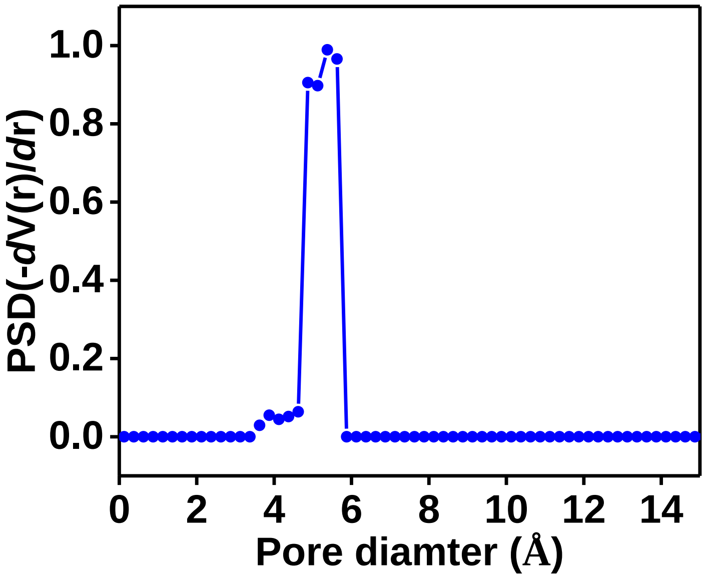

Supplementary Figure 10 | The calculated pore size distribution of 3D-TPE-COF. Based on the crystal structure, the pore size distribution of 3D-TPE-COF was calculated by using Poreblazer[5]. Accordingly, the COF show one pore (0.55 nm), which is consistent with the experiment data.

Supplementary Method 4

Solid-state UV-Vis and Fluorescence Spectra

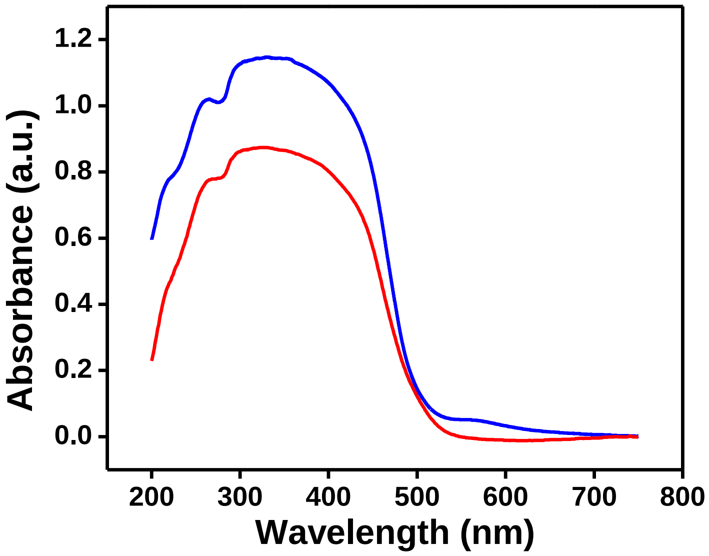

Supplementary Figure 11 | Solid-state UV-Vis spectra of model compound (red curve) and 3D-TPE-COF (blue curve)

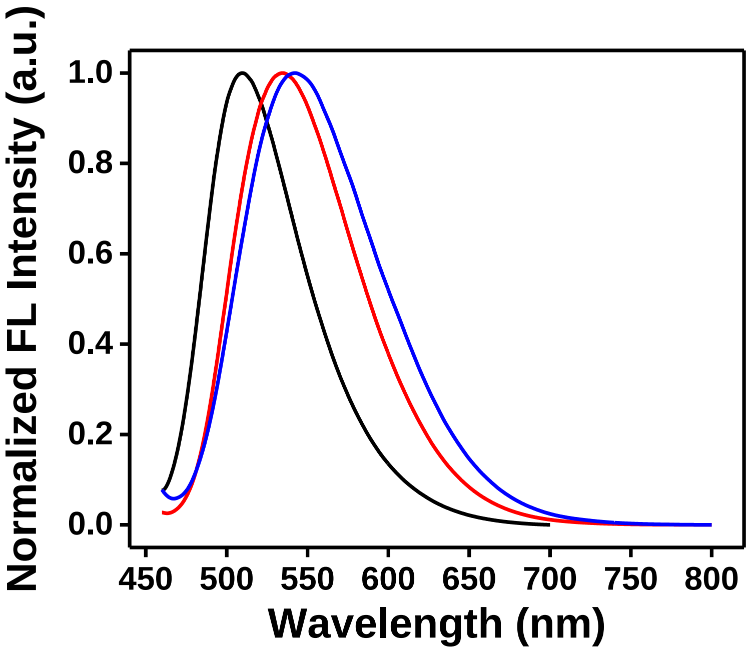

Supplementary Figure 12 | Normalized solid-state fluorescence spectra of TPE-Ph-CHO (black curve), model compound (red curve) and 3D-TPE-COF (blue curve) (λ_{ex} = 450 nm).

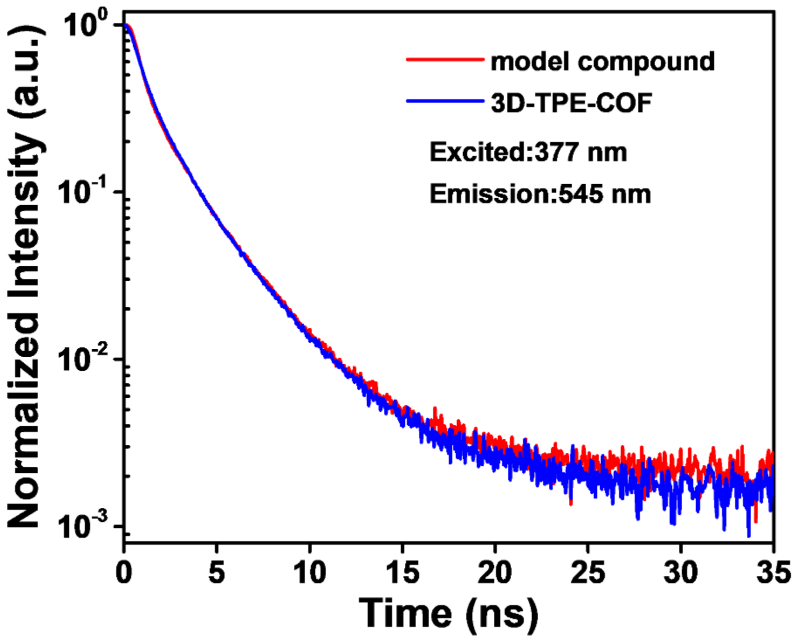

Supplementary Figure 13 | The fluorescence lifetime profile of the model compound (red curve) and 3D-TPE-COF (blue curve). The wavelength of the pumping laser is 377 nm.

Supplementary Method 5

Sensing Study

The powder of 3D-TPE-COF (2.7 mg, 2.56 mmol) was well dispersed in 25 mL water by sonication for 10 min. In a typical experiment, 2 mL suspension was loaded in a quartz cell and then the aqueous solution of picric acid (PA, 1.0×10^{-2} mol L^{-1}) was added gradually. Obviously, the fluorescence of 3D-TPE-COF was quenched when PA was gradually added into the suspension (Supplementary Figure 14). The corresponding fluorescence emission spectra and the fluorescence intensity of the mixture were recorded by excitation at 450 nm.

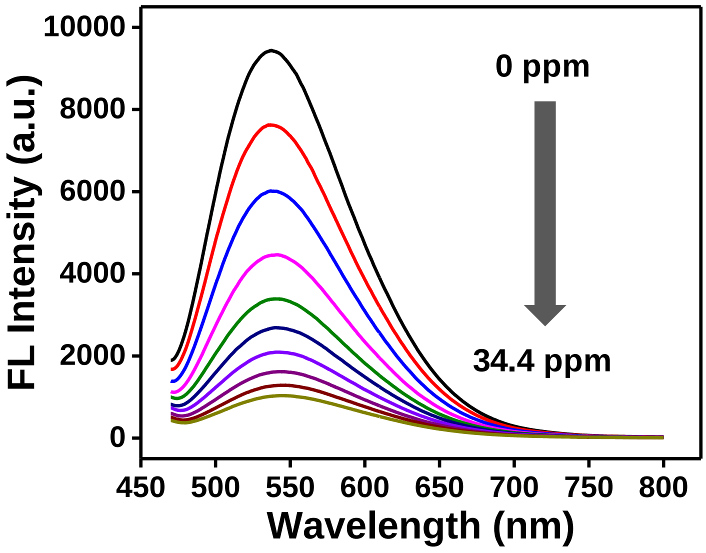

Supplementary Figure 14 | Fluorescence quenching upon addition of PA (0−34.4 ppm) in H_2O (λ_{ex} = 450 nm)

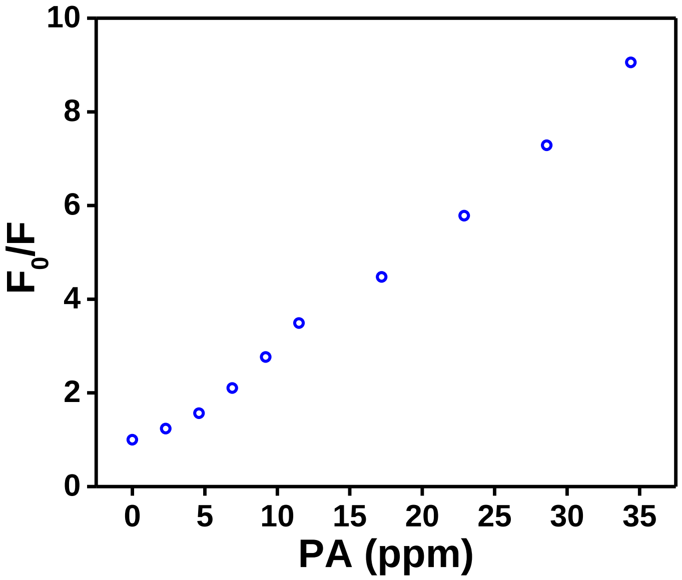

Supplementary Figure 15 | The Stern-Volmer plots obtained from titration of 3D-TPE-COF with PA.

Supplementary Method 6

Fabrication of COF-coated WLEDs

Dip-coating process: In terms of the dip-coating, the blue LED was dipped into the mixture of UV curable epoxy (Norland 63) and 3D-TPE-COF which was homogenously dispersed, and then the device was cured immediately for 2 min after hauling out. This process can be repeated for several times in order to tune the spectrum of the white LED.

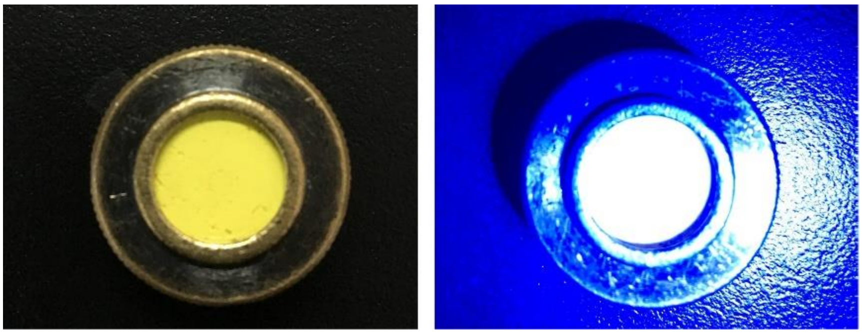

Supplementary Figure 16 | 3D-TPE-COF powder under natural light (left) and blue light (right).

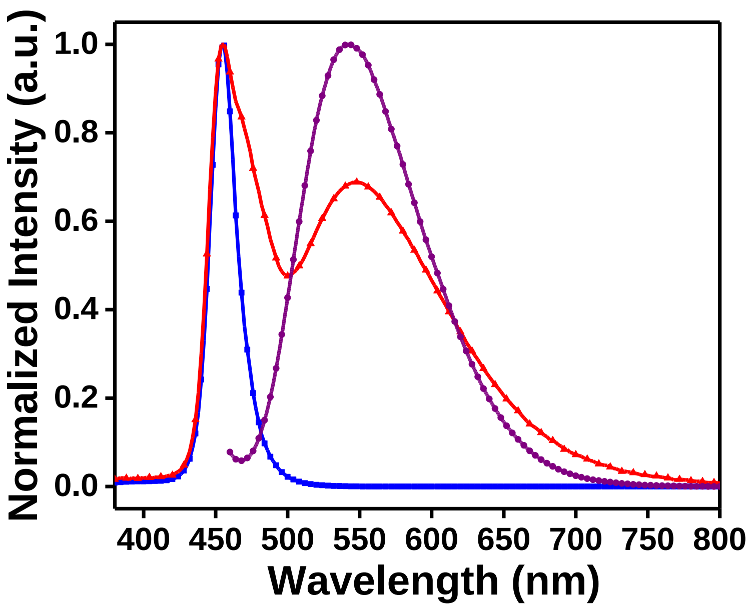

Supplementary Figure 17 | Electroluminescence spectra of the commercial LED (blue) and 3D-TPE-COF coated LED (red), and emission spectrum of 3D-TPE-COF (purple) at room temperature (λ_{ex} = 450 nm).

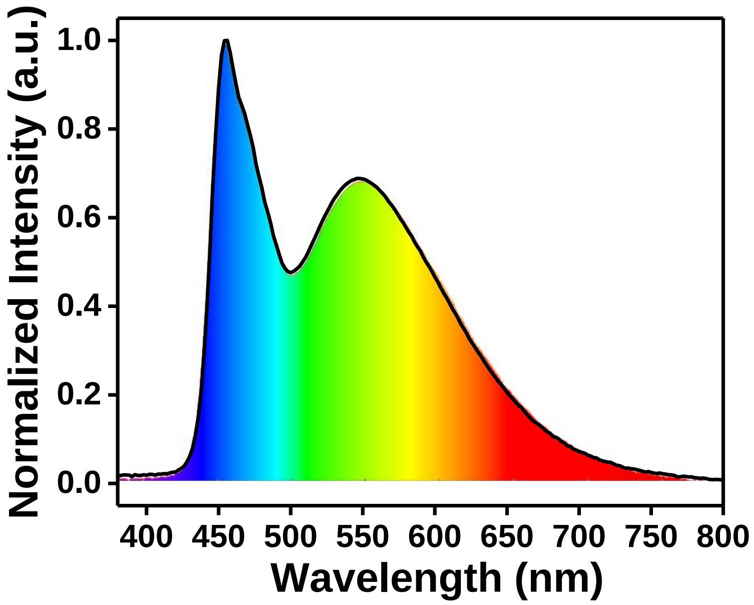

Supplementary Figure 18 | Luminescence spectrum of the 3D-TPE-COF coated WLED with the CIE coordinates of (0.30, 0.35).

Supplementary Figures

^{1}H and ^{13}C NMR Spectra

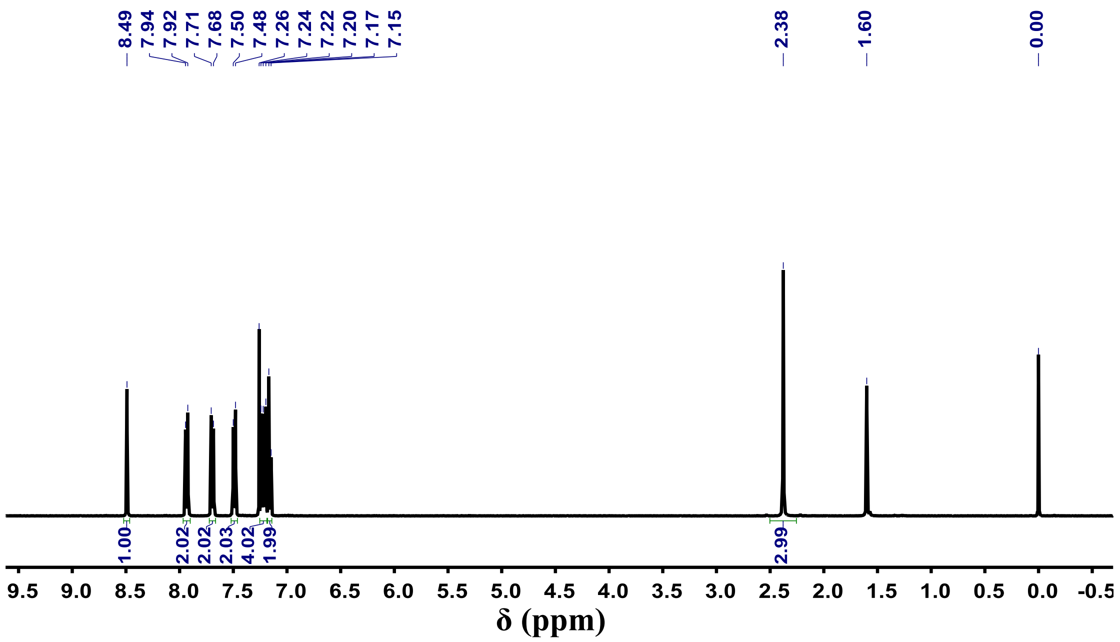

Supplementary Figure 19 | ^{1}H NMR spectrum of model compound in $CDCl_3$ solution.

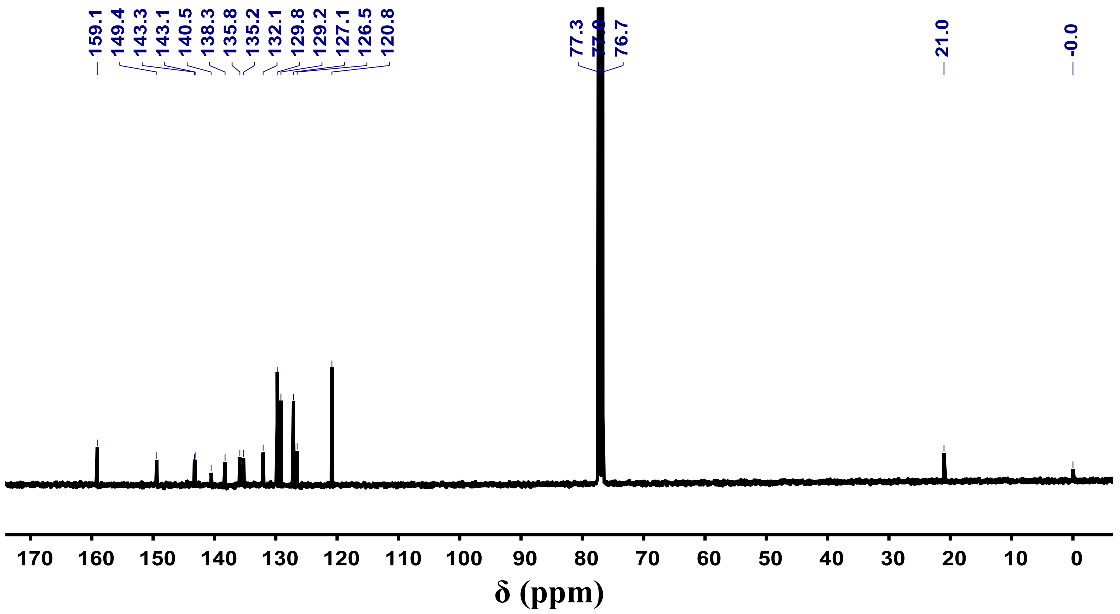

Supplementary Figure 20 | ^{13}C NMR spectrum of model compound in $CDCl_3$

solution.

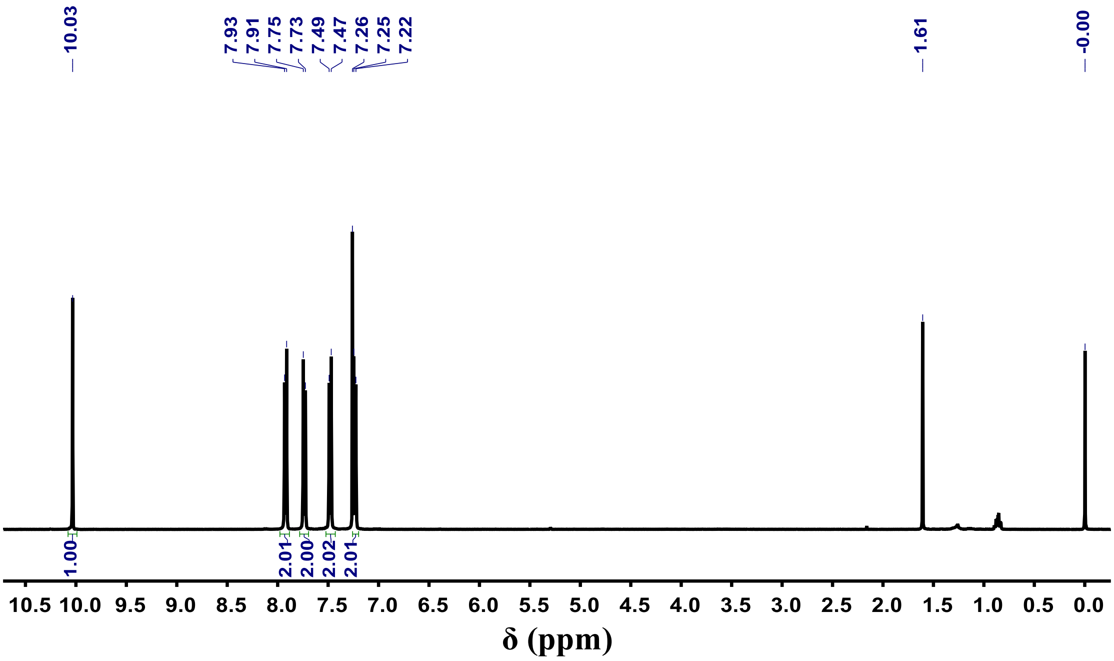

Supplementary Figure 21 | ^{1}H NMR spectrum of TPE-Ph-CHO in $CDCl_3$ solution.

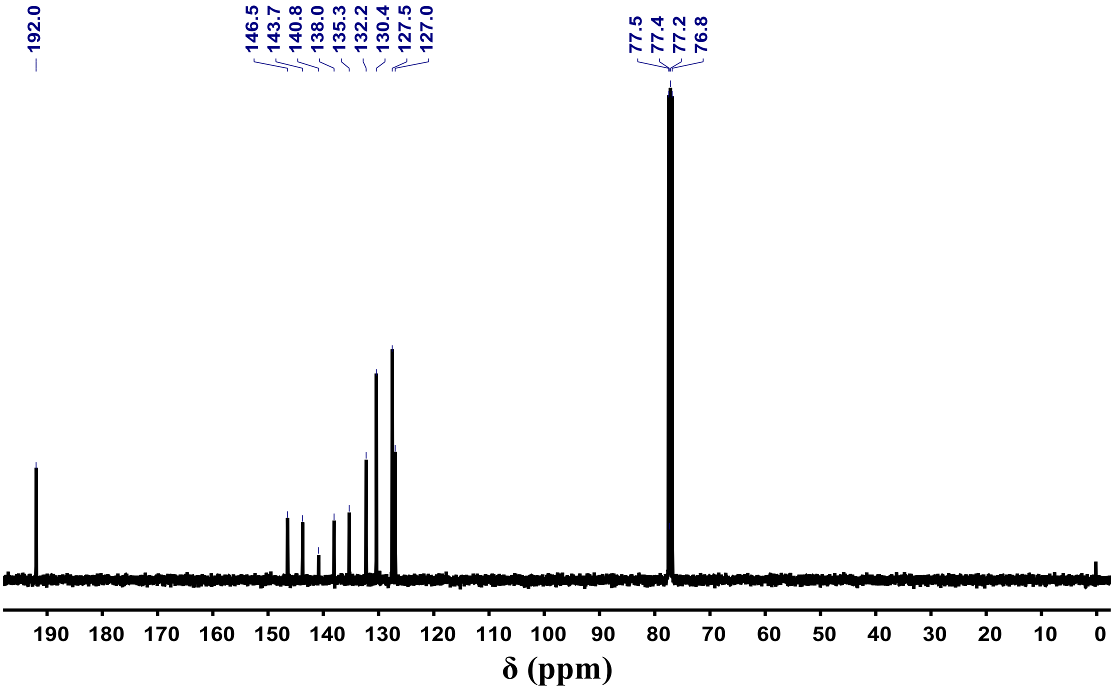

Supplementary Figure 22 | ^{13}C NMR spectrum of TPE-Ph-CHO in $CDCl_3$ solution.

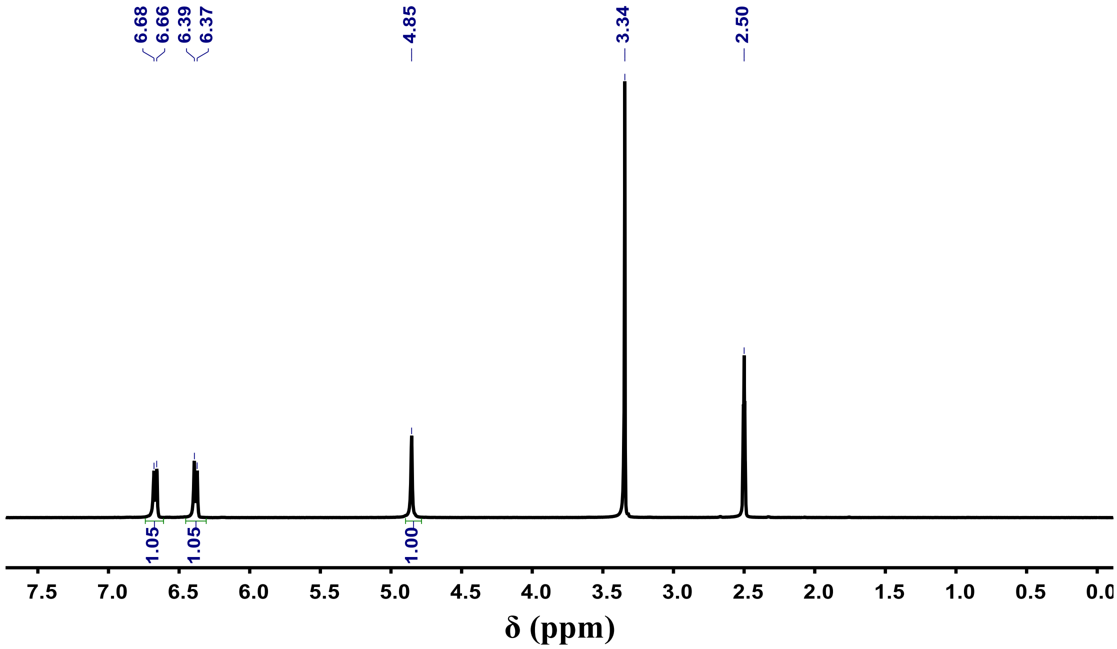

Supplementary Figure 23 | ^{1}H NMR spectrum of TAPM in DMSO-d_6 solution.

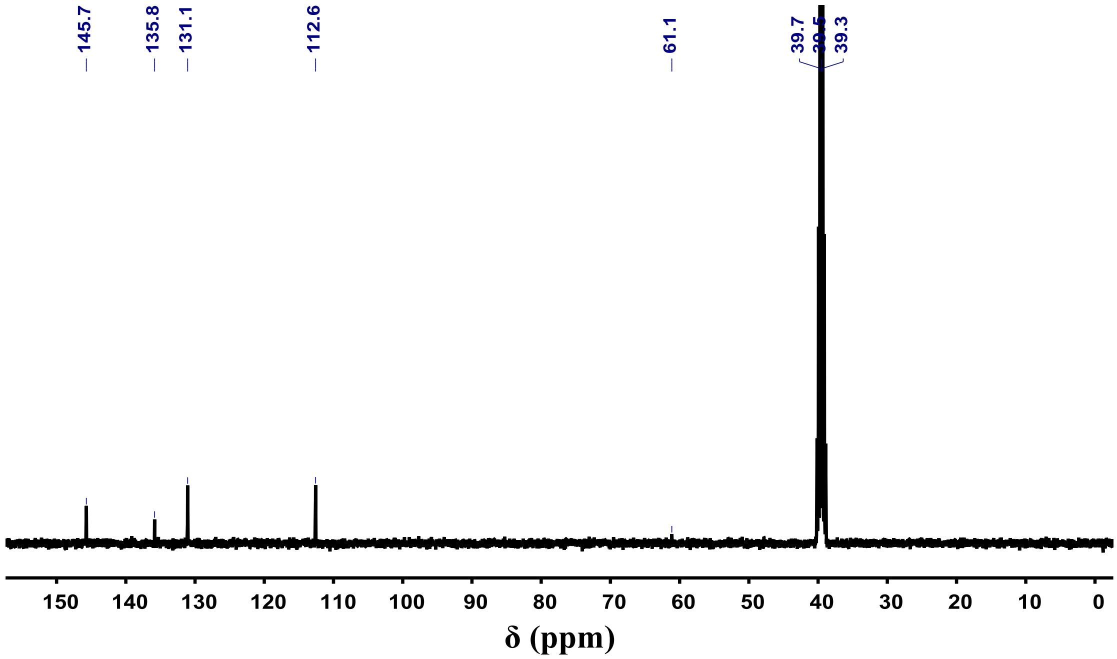

Supplementary Figure 24 | ^{13}C NMR spectrum of TAPM in DMSO-d_6 solution.

Supplementary References

1. Luo, W. *et al.* A dynamic covalent imine gel as a luminescent sensor. *Chem. Commun.* **50**, 11942−11945 (2014).
2. Ganesan, P. et al. Tetrahedral n-type materials: efficient quenching of the excitation of p-type polymers in amorphous films. *J. Am. Chem. Soc.* **127**, 14530–14531 (2005).
3. Wan, W., Sun, J., Su, J., Hovmöller S. & Zou, X. Three-dimensional rotation electron diffraction: software RED for automated data collection and data processing. *J. Appl. Crystallogr.* **46**, 1863–1873 (2013).
4. Sheldrick, G. M. Crystal structure refinement with SHELXL. *Acta Crystallogr. C Struct. Chem.* **A71**, 3–8 (2015).
5. Sarkisov, L. & Harrison A. Computational structure characterization tools in application to ordered and disordered porous materials. *Mol. Simul.* **37**, 1248−1257 (2011).
